# Syphilitic ostial coronary artery occlusion treated with percutaneous coronary intervention: Case series and literature review^☆^

**DOI:** 10.1016/j.ahjo.2023.100337

**Published:** 2023-10-21

**Authors:** Yasmine Baydoun, Yuriy Stukov, Matthew S. Purlee, Bailey Chagnon, Sergiy V. Salo, Andrii Y. Gavrylyshin, Vasyl Lazoryshynets, Olena Levchyshyna, Omar M. Sharaf, Giles J. Peek, Mark S. Bleiweis, Jeffrey P. Jacobs

**Affiliations:** a Congenital Heart Center, Division of Cardiovascular Surgery, Departments of Surgery and Pediatrics, University of Florida, Gainesville, FL, United States of America; b M. M. Amosov National Institute of Cardiovascular Surgery, The National Academy of the Medical Sciences of Ukraine, Kyiv, Ukraine; c Center for Regenerative Medicine, Division of Cardiovascular Medicine, Department of Medicine, University of Florida, Gainesville, FL, United States of America

**Keywords:** Acute coronary syndrome, Coronary artery disease, Percutaneous coronary intervention, Tertiary syphilis

## Abstract

**Introduction:**

Non-atherosclerotic causes of acute coronary syndrome (ACS) are important contributors to a substantial number of acute ischemic coronary events. Syphilitic aortitis is a rare complication of tertiary cardiovascular syphilis that may result in ostial coronary artery stenosis, aortic insufficiency, and ascending aortic aneurysm.

**Methods:**

In this manuscript, we present two Case Reports of patients with bilateral syphilitic coronary artery ostial occlusion, and we review the associated literature. The immunofluorescent test was positive for syphilis in both patients.

**Results:**

Diagnostic coronary angiography revealed bilateral occlusions of the left main coronary artery (LMCA) and right coronary artery (RCA), which were successfully treated with percutaneous coronary intervention (PCI) with bare metal stents (BMS). After deployment of the stents, arterial blood flow was re-established with TIMI flow grade 3.

**Discussion:**

The angiographic finding of bilateral coronary ostial lesions in young patients should raise the suspicion of cardiovascular syphilis. Options for revascularization should be discussed amongst the patient and the Heart Team. PCI may be an option for treatment of isolated syphilitic coronary stenosis in the setting of acute hemodynamic instability or chronic inflammation.

## 1 Introduction

The global annual incidence of syphilis is estimated to be approximately 6 million cases per year in patients aged 15 to 49 years [1], which poses a serious threat to public health. Despite ongoing efforts to improve the diagnosis and treatment of syphilis, occasional cases of untreated chronic syphilis occur, especially in developing countries. Syphilitic cardiovascular disease occurs more frequently than it is recognized clinically [2]. Cardiovascular syphilis may lead to aortitis. Among patients with untreated syphilis, aortitis occurs in up to 70%–80% of these patients, and clinically apparent manifestations of aortic regurgitation, coronary arterial ostial stenosis and aortic aneurysms are seen in 10%–15% of these patients [3]. Indeed, cardiovascular manifestations of tertiary syphilis can include aortitis, aortic root dilation, aortic aneurysm formation, aortic insufficiency, and coronary arterial ostial stenosis. Importantly, coronary arterial ostial lesions have been detected in as many as 26% of patients with syphilitic aortitis [4]. In this manuscript, we describe two patients treated in Ukraine with bilateral coronary ostial stenosis secondary to tertiary syphilis, and we review the associated literature.

## 2 Case 1

A 37-year-old female was transferred to the coronary reperfusion center from an outside hospital within the therapeutic window for an ST-Segment Elevation Myocardial Infarction (STEMI). The patient did not have any previous medical history and did not have any cardiovascular risk factors. Physical examination revealed a heart rate of 90 beats per minute and blood pressure of 100/60 mm Hg. Electrocardiography revealed ST elevations in leads aVR, V2, and V3; ST depressions in leads II, III, aVF, V4, V5, and V6; and a PQ interval of 0.16 s. Emergent echocardiography revealed moderate aortic regurgitation, mild mitral regurgitation, and an ejection fraction of 60%. Laboratory work up documented the following values: CK-MB = 19 U/L, CPK = 41 U/L, LDH = 169 U/L, AST = 27 U/L, and ALT = 28 U/L. Although no cutaneous lesions were observed, the patient had laboratory evidence of syphilis, as documented by the following values: rapid plasma reagin (RPR) = 1:32, Wassermann test (WT) = 1:80, and a positive immunofluorescence assay.

**Fig. 1:**
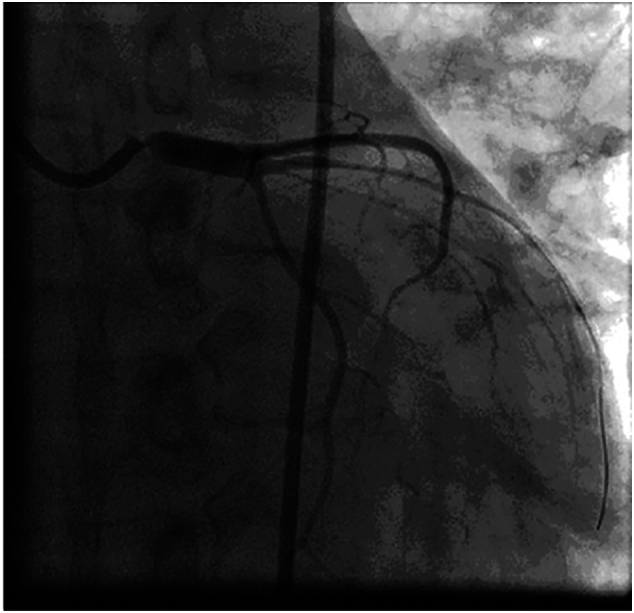
Case #1. Coronary angiography revealed subtotal ostial stenosis of the LMCA.

**Fig. 2:**
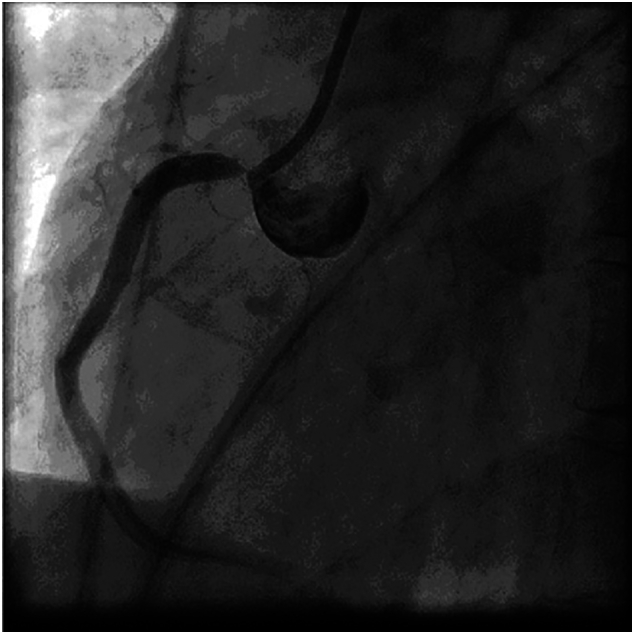
Case #1. Coronary angiography revealed subtotal stenosis of the RCA.

**Fig. 3:**
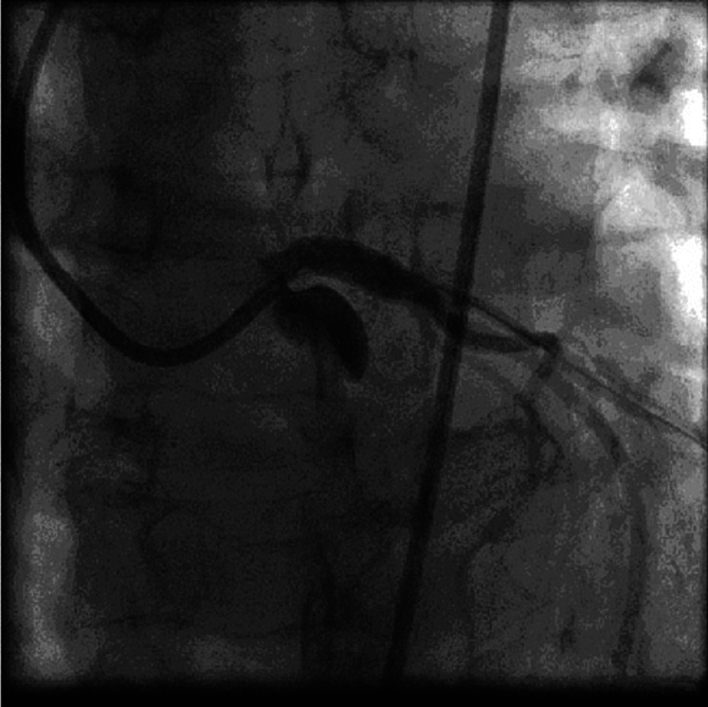
Case #1. Angiography of the LMCA after BMS placement. The LMCA was revascularized with a 4.0 × 15.0 CID (Chrono cobalt‑chromium alloy carbofilm-coated coronary stent) system, deployed with 16 atm pressure.

**Fig. 4:**
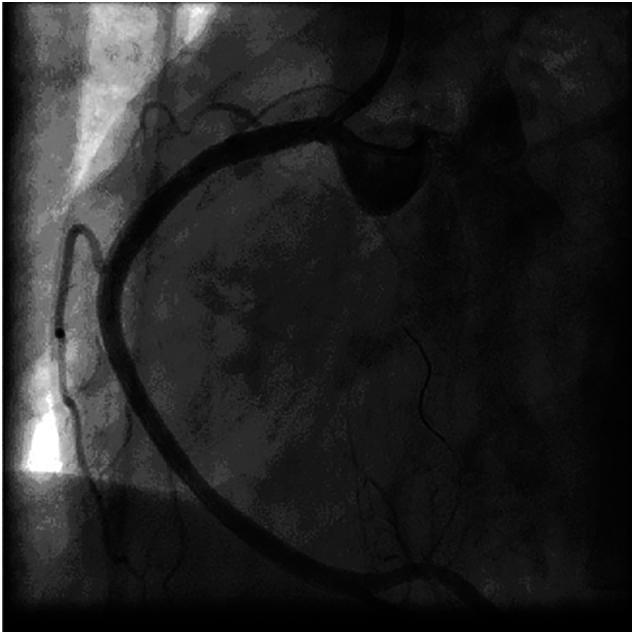
Case #1. Angiography of the RCA after BMS placement. The RCA was revascularized with a 3.5 × 16.0 CID (Chrono cobalt‑chromium alloy carbofilm-coated coronary stent) system, deployed with 18 atm pressure.

Emergent coronary angiography (Fig. 1, Fig. 2) revealed 95%–100% subtotal ostial stenosis of the left main coronary artery (LMCA) and 95% ostial occlusion of the right coronary artery (RCA). Due to the hemodynamic instability of the patient, the Heart Team decided to proceed with emergent percutaneous coronary intervention (PCI). Two bare metal stents (BMS's) were placed (Fig. 3, Fig. 4), one in the LMCA and one in the proximal RCA. Coronary flow was restored in both arteries with TIMI flow grade 3. The LMCA was revascularized with a 4.0 × 15.0 CID (Chrono cobalt‑chromium alloy carbofilm-coated coronary stent) system, deployed with 16 atm pressure. The RCA was revascularized with a 3.5 × 16.0 CID (Chrono cobalt‑chromium alloy carbofilm-coated coronary stent) system, deployed with 18 atm pressure. The patient was started on aspirin, clopidogrel, atorvastatin, and penicillin. During the hospital stay, the patient remained hemodynamically stable and asymptomatic.

After medical stabilization, the patient underwent additional evaluation and treatment for tertiary syphilis. The patient was subsequently evaluated 10 years after PCI and was free of symptoms, with moderate physical tolerance. The patient has not required any additional coronary interventions.

## 3 Case 2

A 54-year-old male presented to his primary care provider complaining of moderate dyspnea after infection with coronavirus disease 2019 (COVID-19). The COVID-19 infection resolved spontaneously; however, two months later, the patient experienced recurrent episodes of dyspnea. The patient was then referred for evaluation by a cardiologist.

Angiography revealed multi-vessel coronary artery disease and moderate aortic insufficiency. The patient had elevated RPR titers as well as a positive *Treponema pallidum* particle agglutination assay (TP-PA). The patient was treated with a course of penicillin for tertiary syphilis, and then the patient was transferred to our Center for revascularization.

**Fig. 5:**
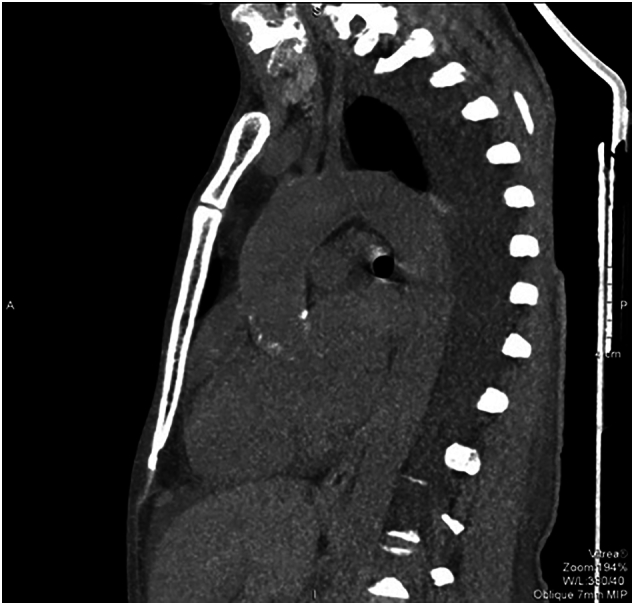
Case #2. A non-contrast computerized axial tomographic scan revealed signs of aortitis, with calcification of the aortic root and bilateral hydrothorax. Of note, the aortic wall is thickened and aortic root calcinosis is seen.

**Fig. 6:**
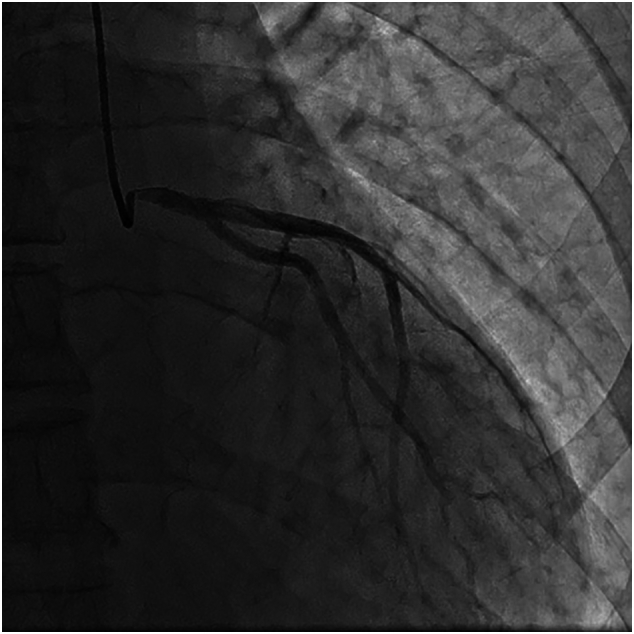
Case #2. Coronary angiography revealed 75% ostial stenosis of the LMCA.

**Fig. 7:**
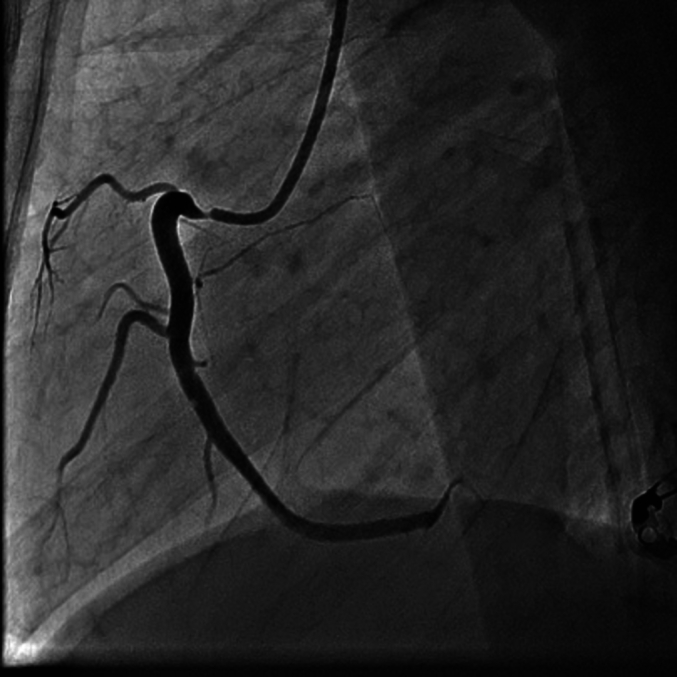
Case #2. Coronary angiography revealed 90% ostial stenosis of the RCA.

A non-contrast computerized axial tomographic scan revealed signs of aortitis, with calcification of the aortic root and bilateral hydrothorax (Fig. 5). Coronary angiography revealed bilateral subtotal ostial stenosis of the LMCA (Fig. 6) and the RCA (Fig. 7).

**Fig. 8:**
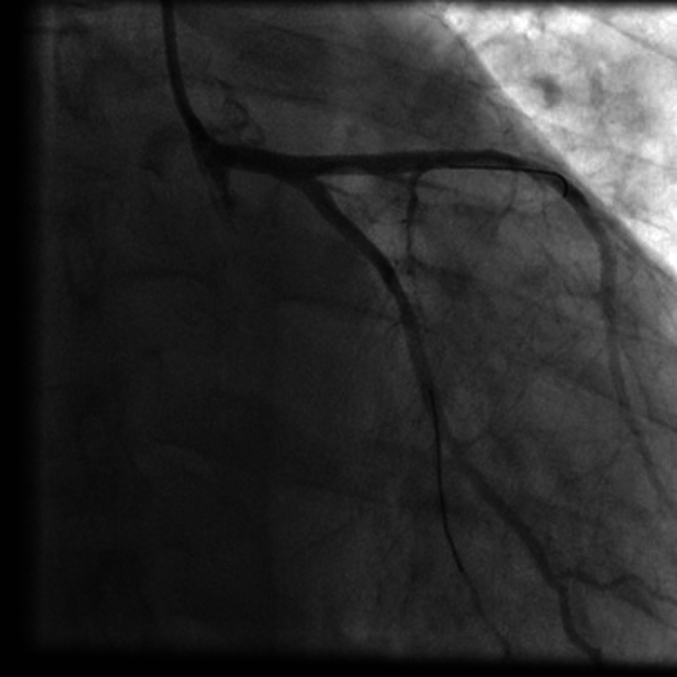
Case #2. Angiography of the LMCA after placement of DES. The LMCA was revascularized with a 4 × 14 DES, deployed with 18 atm pressure.

**Fig. 9:**
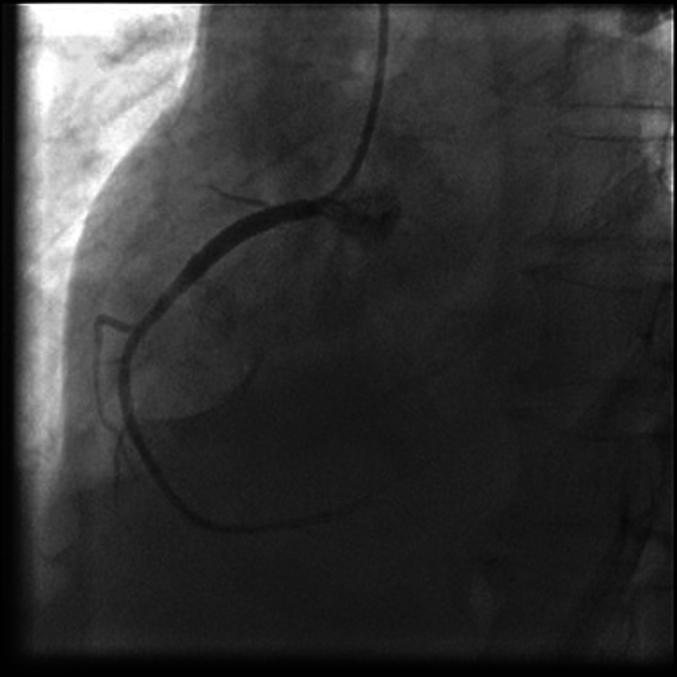
Case #2. Angiography of the RCA after placement of BMS. The RCA was revascularized with a 4 × 29 BMS, deployed with 18 atm pressure.

The patient underwent PCI with placement of two stents: one stent in the ostial lesion of the LMCA and one stent in the ostial lesion of the RCA. These stents were placed via the right radial artery, using a 6 French sheath. The LMCA was revascularized with a 4 × 14 drug eluting stent (DES), deployed with 18 atm pressure (Fig. 8). The RCA was revascularized with a 4 × 29 BMS, deployed with 18 atm pressure (Fig. 9). After coronary stenting, arterial blood flow was re-established with TIMI flow grade 3.

## 4 Discussion

Cardiovascular syphilis typically occurs 10 to 40 years after primary syphilitic infection and is seen in 10% of all patients with tertiary syphilis. Syphilitic aortitis is inflammation of the aorta that is associated with tertiary syphilis. Syphilitic aortitis may be associated with ostial coronary artery stenosis.

Tewari and Moorthy [4] reported the case of a 44-year-old man with unstable angina and no cardiovascular risk factors who had an ulcer over his scrotum 20 years back. Electrocardiography revealed ST-Segment depression in the anterolateral and inferior leads and ST-Segment elevation in aVR. Echocardiography showed aneurysmal ostial dilation of the RCA. Coronary angiography identified critical stenosis of the LMCA and aneurysmal ostial dilation of the RCA. Blood serology was positive for both VDRL and TPHA.

Tertiary syphilis may also cause local changes in the heart muscle and can be associated with myocardial gummas [5], coronary arterial stenoses and aneurysms, aortic valvar insufficiency, and thoracic aortic aneurysm. As described above, aortitis occurs in up to 70%–80% of patients with untreated syphilis, and clinically apparent manifestations of aortic regurgitation, coronary arterial ostial stenosis and aortic aneurysms are seen in 10%–15% of these patients [3]. In general, ascending thoracic aneurysms can be associated with [[6], [7], [8]]:

• noninflammatory/degenerative aortic diseases with degeneration of the aortic media, or
• inflammatory diseases of the aorta including atherosclerosis and aortitis.

The differential diagnosis of isolated ascending aortitis includes syphilitic aortitis as well as rheumatological conditions including Takayasu's disease, and giant cell aortitis [9].

Coronary ostial lesions may appear in approximately 26% of patients with syphilitic aortitis [4]. Weakening of the aortic wall can lead to aortic root dilation and aortic regurgitation, and can also lead to formation of aortic aneurysms. Aortic wall thickening and fibrosis can lead to stenosis of the ostium(s) of the coronary arteries [10]. With syphilis complicated by stenosis of the coronary arteries, anginal symptoms are experienced frequently, but myocardial infarction rarely develops. This feature can be attributed to the relatively slow narrowing of the ostium(s) of the coronary arteries, creating favorable conditions for the development of collateral circulation. Nevertheless, cases of sudden death have been reported in patients with syphilis.

Syphilitic aortitis is defined as inflammation of the aorta in the setting of syphilis. Syphilitic aortitis typically involves the ascending aorta and typically occurs in the setting of tertiary syphilis. In the initial phases of tertiary syphilis, clinically asymptomatic inflammation of the ascending aorta may develop. The eventual diagnosis for syphilitic aortitis greatly depends on the clinical manifestations present. If a patient develops syphilitic aortitis, sacculated aneurysms and coronary ostial stenosis are the most common manifestations [11]. On physical examination, a large syphilitic aortic aneurysm may be noted as an ectopic pulsatile impulse in the right upper chest, while aortic insufficiency may initially be diagnosed on auscultation with a stethoscope.

In tertiary syphilis, the lesion of the coronary arteries is comparable to the occurrence of obliterating endoarteritis, in that a specific infiltrate develops in the ostium of the arteries, creating ideal conditions for coronary spasm. In contrast to atherosclerosis, the infiltrate does not spread along the coronary arteries, but is limited to the initial area, resulting in ostial stenosis, which can eventually lead to the development of acute coronary syndrome.

Based on these facts, evaluation of the serum of a patient for syphilis should be considered in the following clinical scenarios:

• young patients without cardiac risk factors who develop acute coronary syndrome, and
• patients with aortic regurgitation in association with ostial coronary arterial stenosis.

Consequently, this report of these two cases along with this review of the literature should therefore serve as an important reminder that the development of acute coronary syndrome in a young patient without cardiac risk factors should raise the suspicion of cardiovascular syphilis.

## 5 Conclusions

Angiographic findings of bilateral coronary ostial lesions in young patients should raise the suspicion of cardiovascular syphilis. Options for revascularization should be discussed amongst the patient and the Heart Team. PCI may be an option for treatment of isolated syphilitic coronary stenosis in the setting of acute hemodynamic instability or chronic inflammation.

## Funding

No funding source was used in the writing or submission of this manuscript.

## Consent to participate/publication

Consent for these two Case Reports were obtained from the two patients.

## Submission declaration/verification

We confirm that these Case Reports have not been submitted to other journals.

## Ethical standards

The authors assert that all procedures contributing to this work comply with the ethical standards of the relevant national guidelines on human experimentation and with the Helsinki Declaration of 1975, as revised in 2008.

## Declaration of competing interest

The authors declare that they have no known competing financial interests or personal relationships that could have appeared to influence the work reported in this paper.
